# Bacteriology of peritonsillar abscess: the changing trend and predisposing factors^☆^

**DOI:** 10.1016/j.bjorl.2017.06.007

**Published:** 2017-07-17

**Authors:** Yi-Wen Tsai, Yu-Hsi Liu, Hsing-Hao Su

**Affiliations:** a Kaohsiung Veterans General Hospital, Department of Medical Education and Research, Kaohsiung, Taiwan; b Kaohsiung Veterans General Hospital, Department of Otorhinolaryngology, Head and Neck Surgery, Kaohsiung, Taiwan; c Tajen University, Department of Pharmacy and Graduate Institute of Pharmaceutical Technology, Pingtung, Taiwan

**Keywords:** Anaerobic bacteria, Bacterial infections, *Klebsiella pneumoniae*, Peritonsillar abscess, *Viridans streptococci*, Bactérias anaeróbicas, Infecções bacterianas, *Klebsiella pneumoniae*, Abscesso peritonsilar, *Viridans streptococci*

## Abstract

**Introduction:**

Peritonsillar abscess is the most common deep neck infection. The infectious microorganism may be different according to clinical factors.

**Objective:**

To identify the major causative pathogen of peritonsillar abscess and investigate the relationship between the causative pathogen, host clinical factors, and hospitalization duration.

**Methods:**

This retrospective study included 415 hospitalized patients diagnosed with peritonsillar abscess who were admitted to a tertiary medical center from June 1990 to June 2013. We collected data by chart review and analyzed variables such as demographic characteristics, underlying systemic disease, smoking, alcoholism, betel nut chewing, bacteriology, and hospitalization duration.

**Results:**

A total of 168 patients had positive results for pathogen isolation. *Streptococcus viridans* (28.57%) and *Klebsiella pneumoniae* (23.21%) were the most common microorganisms identified through pus culturing. The isolation rate of anaerobes increased to 49.35% in the recent 6 years (*p* = 0.048). Common anaerobes were *Prevotella* and *Fusobacterium* spp. The identification of *K*. *pneumoniae* increased among elderly patients (age > 65 years) with an odds ratio (OR) of 2.76 (*p* = 0.03), and decreased in the hot season (mean temperature > 26 °C) (OR = 0.49, *p* = 0.04). No specific microorganism was associated with prolonged hospital stay.

**Conclusion:**

The most common pathogen identified through pus culturing was *S. viridans*, followed by *K*. *pneumoniae*. The identification of anaerobes was shown to increase in recent years. The antibiotics initially selected should be effective against both aerobes and anaerobes. Bacterial identification may be associated with host clinical factors and environmental factors.

## Resumo

**Introdução:**

O Abscesso Peritonsilar é a infecção cervical profunda mais comum. O microrganismo infeccioso pode ser diferente de acordo com os fatores clínicos.

**Objetivo:**

Identificar o principal agente causador do abscesso peritonsilar e investigar a relação entre o patógeno causador, os fatores clínicos do hospedeiro e a duração da hospitalização.

**Método:**

Este estudo retrospectivo incluiu 415 pacientes hospitalizados diagnosticados com abscesso peritonsilar que foram internados em um centro médico terciário de junho de 1990 a junho de 2013. Coletamos dados através da análise dos arquivos médicos dos pacientes e analisamos variáveis como características demográficas, doença sistêmica subjacente, tabagismo, alcoolismo, hábito de mascar noz de betel, bacteriologia e duração da hospitalização.

**Resultados:**

Um total de 168 pacientes apresentaram resultados positivos para isolamento de patógenos. *Streptococcus viridans* (28,57%) e *Klebsiella pneumoniae* (23,21%) foram os microrganismos mais comuns identificados pela cultura da secreção. A taxa de isolamento de anaeróbios aumentou para 49,35% nos últimos 6 anos (*p* = 0,048). Os anaeróbios comuns foram *Prevotella* e *Fusobacterium spp*. A identificação de *K. pneumoniae* aumentou em pacientes idosos (idade > 65 anos) com razão de chances (*Odds Ratio* - OR) de 2,76 (*p* = 0,03) e diminuiu na estação do calor (temperatura média > 26 °C) (OR = 0,49, *p* = 0,04). Nenhum microrganismo específico foi associado à hospitalização prolongada.

**Conclusão:**

O patógeno mais comumente identificado através da cultura de secreção foi *S. viridans*, seguido por *K. pneumoniae*. A identificação de anaeróbios mostrou ter aumentado nos últimos anos. Os antibióticos selecionados inicialmente devem ser efetivos contra aeróbios e anaeróbios. A identificação bacteriana pode estar associada a fatores clínicos e fatores ambientais do hospedeiro.

## Introduction

Peritonsillar abscess (PTA), or quinsy, is the most common deep neck infection.^1^ The abscess may spread into the parapharyngeal space of other deep neck spaces, to the adjacent structure, and to the bloodstream. It rarely occurs but PTA is potentially life threatening. Early diagnosis of PTA is extremely crucial, and appropriate antibiotics and surgical intervention to remove the abscess are required.^2^ Antibiotics result in a substantial reduction in the progression of this disease. The empirical antibiotic used should be effective against the possible causative pathogen of PTA.

Our objectives were to investigate the microbiology of PTA and to identify its relationship with clinical variables including the underlying systemic disease of patients; habits such as smoking, alcoholism, and betel nut chewing; and hospitalization duration.

## Methods

### Study design and sample population

This retrospective study included 415 patients with PTA who were admitted to a tertiary medical center located in Southern Taiwan from June 1990 to June 2013. Inclusion criteria were hospitalized patients who were clinically diagnosed with PTA (ICD-9 code 475) by positive pus aspiration or computed tomography (CT) imaging. We reviewed the chart of each patient to collect the following data: admission date, age, sex, height, weight, host clinical factors (diabetes mellitus [DM], hypertension, smoking habit, alcoholism, and betel nut chewing), pus culture result, antibiotic treatment, surgery, and hospitalization duration. The study was approved by the institutional review board.

We classified the bacteria into different categories according to the characteristics of Gram staining and anaerobic properties. We defined prolonged hospitalization as hospitalization duration of more than 6 days. Obesity was defined as a body mass index of more than 27, and elderly patients were defined as those aged older than 65 years. We defined the hot season as the months from May to October when the average temperature in Southern Taiwan was above 26 °C according to the record of the Central Weather Bureau of R.O.C.

### Statistical analysis

All data were analyzed using the SPSS statistical software (IBM Corp., Armonk, NY, USA), except for the Cochran–Armitage test, which was performed using the SAS program (SAS Institute, Cary, NC, USA). The association with each independent variable was statistically analyzed among the different groups. Categorical variables were compared using the Pearson's Chi-square test or the Fisher's exact test, as appropriate. Odds ratios (ORs) and their 95% confidence intervals (CIs) were calculated. Trends of isolated pathogens were analyzed using the Cochran–Armitage test. A *p*-value less than 0.05 was considered statistically significant.

### Ethic statement

This study has been approved by the Institutional Review Board; the approval protocol number is VGHKS14-CT7-01.

## Results

### Demographraphic characteristics

This study included 415 patients. The results of pus cultures from either surgery or needle aspiration were available for 266 patients. Adjustments for sample submitted to tonsil surgery or PTA drainage was performed, as shown in Table 1. There is no patient with history of AIDS or HIV infection in this study.

**Table 1:** Demographraphic characteristics of patients with peritonsillar abscess.

Age	Overall, (*n* = 266)	Diabetes mellitus	Smoking	Alcoholism	Betel-nut chewing
<18 y/o	22 (8.27)	0	6 (27.27)	5 (22.73)	2 (9.09)
18–64 y/o	215 (80.83)	19 (8.84)	111 (51.63)	76 (35.35)	37 (17.21)
≥65 y/o	29 (10.90)	4 (13.79)	5 (17.24)	7 (24.14)	3 (10.34)

Total	266 (100)	23 (8.65)	122 (45.86)	88 (33.08)	42 (15.79)

Data are presented as *n* (%).

y/o indicates “year old”.

### Bacteriology

Within these patients with pus obtained, 230 (230–266, 86.47%) showed bacterial growth in their pus culture. The pus culture of the remaining 36 patients showed no bacterial growth. Of the 230 patients, 132 (132–230, 57.39%) had polymicrobial pus, including 62 cases merely reported as “normal flora” or “mixed flora” (62–230, 26.96%). Pus cultures of 168 patients (168–266, 63.15%) showed positive results for pathogen isolation. More than a single pathogen was isolated in 64 patients (64–168, 38.10%). Aerobic bacteria were isolated from 85.7% (144/168) of positive cultures, anaerobic or facultative aerobic bacteria from 44.0% (74–168), and mixed aerobic and anaerobic bacteria from 29.8% (50–168).

**Figure 1:**
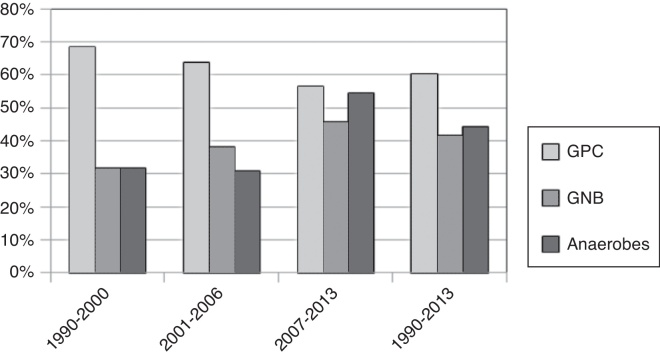
Isolation rate of different types of bacteria during each 6 year interval.

The most common pathogen identified through pus culturing was *Streptococcus viridans* (48–168, 28.57%), followed by *Klebsiella pneumoniae* (39–168, 23.21%) and the beta-hemolytic *Streptococcus* group (17–168, 10.12%), as shown in Table 2. We divided patients by the 4 periods of 1990–1995, 1996–2001, 2002–2007, and 2008–2013; the isolation rate of the anaerobes was 25%, 23.81%, 45.45%, and 49.35%, respectively. The isolation rate of anaerobic pathogens increased significantly between 1990 and 2013 (Cochran–Armitage test, *p* = 0.048), as shown in Table 3 and Fig. 1. The isolation rates of gram-positive bacteria and gram-negative bacteria in these 4 periods were 100% and 25%, 57.14% and 47.62%, 62.12% and 48.48%, and 51.95% and 49.35%, respectively. Most of the anaerobic pathogens were *Prevotella* spp. (24–168, 14.29%) and *Fusobacterium* spp. (16–168, 9.52%), as shown in Table 2.

**Table 2:** Bacteriology of 168 patients with peritonsillar abscess with definite isolation of pus culture.

Causative pathogen	Overall (*n* = 168)	DM (*n* = 17)	HTN (*n* = 22)	Smoking (*n* = 78)	Alcoholism (*n* = 59)	Betel-Nut chewing (*n* = 27)	Obesity (*n* = 30)
Aerobic GNB	51 (30.36)	10 (58.82)	8 (36.36)	22 (28.21)	15 (25.42)	8 (29.63)	8 (26.67)
*Klebsiella pneumoniae*	39 (23.21)	7 (41.18)	7 (31.82)	19 (24.36)	13 (22.03)	7 (25.93)	7 (23.33)
Aerobic GPB	22 (13.10)	10 (58.82)	5 (22.73)	12 (15.38)	11 (18.64)	4 (14.81)	6 (20.00)
Aerobic GNC	4 (2.38)	1 (5.88)	1 (4.55)	1 (1.28)	1 (1.69)	1 (3.70)	2 (6.67)
Aerobic GPC	99 (58.93)	10 (58.82)	12 (54.55)	46 (58.97)	32 (61.02)	21 (77.78)	19 (63.33)
*Staphylococcus* spp.	9 (5.36)	0 (0.00)	1 (4.55)	5 (4.6)	5 (8.47)	1 (3.70)	2 (6.67)
Beta-hemolytic *streptococcus* group	18 (10.71)	1 (5.88)	1 (4.55)	15 (19.23)	10 (16.95)	4 (14.81)	4 (13.33)
*Streptococcus* melliri group	24 (14.29)	4 (23.53)	4 (18.18)	10 (12.82)	8 (13.56)	9 (33.33)	4 (13.33)
*Streptococcus viridans* group	48 (28.57)	5 (29.41)	7 (31.82)	16 (20.51)	13 (22.03)	7 (25.93)	7 (23.33)
Anaerobic cocci	34 (20.23)	5 (29.41)	5 (22.73)	17 (21.79)	13 (22.03)	7 (25.93)	11 (36.67)
*Peptostreptococcus* spp.	9 (5.36)	1 (5.88)	1 (4.55)	7 (8.97)	6 (10.17)	3 (11.11)	4 (13.33)
Anaerobic GPB	14 (8.33)	1 (5.88)	5 (22.73)	5 (6.41)	4 (6.78)	2 (7.41)	3 (10.00)
Anaerobic GNB	45 (26.79)	1 (5.88)	6 (27.27)	24 (30.77)	18 (30.51)	7 (25.93)	6 (20.00)
*Fusobacterium* spp.	17 (10.12)	0 (0.00)	1 (4.55)	10 (12.82)	6 (10.17)	3 (11.11)	2 (6.67)
*Prevotella* spp.	24 (14.29)	1 (5.88)	4 (18.18)	11 (14.10)	10 (16.95)	4 (18.18)	4 (13.33)

Data are presented as *n* (%).

Aerobic isolates included aerobic and facultative anaerobic isolates.

DM, diabetes mellitus; GNB, gram-negative bacilli; GNC, gram-negative cocci; GPB, gram-positive bacilli; GPC, gram-positive cocci; HTN, hypertension.

**Table 3:** Isolation rate of different types of bacteria during each 6 year interval, 1990–2013.

Years, number of patient types (%) of bacteria	1990–1995	1996–2001	2002–2007	2008–2013	Total (1990–2013)	Test for trends (*p*-value)
Gram positive (% of total patient)	4 (100)	12 (57.14)	41 (62.12)	40 (51.95)	97 (57.74)	0.120
Gram negative (% of total patient)	1 (25)	10 (47.62)	32 (48.48)	38 (49.35)	81 (48.21)	0.569
Anaerobes (% of total patient)	1 (25)	5 (23.81)	30 (45.45)	38 (49.35)	74 (44.05)	0.048^a^
Patient	4	21	66	77	168	

a Denotes for *p*-value less than 0.05.

Host clinical factors were associated with several isolated pathogens. Betel nut chewing was associated with the isolation of gram-positive cocci (GPC) (OR = 2.67, *p* = 0.04). The association of bacterial isolation with smoking habit and alcoholism was not statistically significant. Elderly patients (age > 65 years) had higher *K. pneumoniae* isolation (OR = 2.76, *p* = 0.03). Obesity (BMI > 27) was associated with a higher isolation of *Peptostreptococcus* (OR = 4.19, *p* = 0.04), as shown in Table 4.

**Table 4:** Association between the predisposing factors and the pathogen.^a^

Predisposing factors	Causative pathogen	OR	95% CI	*p*-value
Elder^b^	KP	2.76	1.10–6.93	0.03^e^
Obesity^c^	*Peptostreptococcus*	4.19	0.98–17.88	0.04^e^
Hot season^d^	GPB	3.22	1.13–9.19	0.02^e^
	KP	0.49	0.23–1.01	0.04^e^
Betel-Nut chewing	GPC	2.67	1.02–7.02	0.04^e^

a No statistically difference was observed among bacterial isolates and smoking, alcoholism, and DM.

b Elderly indicates patient's age was more than 65 years old.

c Obesity indicates patient's body mass index was more than 27.

d Hot season indicates the admission date was between May and October, during which time the average temperature in southern Taiwan was more than 27 °C.

e Denotes for *p*-value less than 0.05. CI, confidence interval; DM, diabetes mellitus; GPB, gram-positive bacilli; GPC, gram-positive cocci; HTN, hypertension; KP, *Klebsiella pneumoniae*; OR, odds ratio.

In addition, in the hot season, we found that the risk of isolating gram-positive bacilli (GPB) increased (OR = 3.22, *p* = 0.02), but that of *K. pneumoniae* isolation decreased (OR = 0.49, *p* = 0.04), as shown in Table 4. There was no specific microorganism associated with prolonged hospital stay.

Searching from the PubMed database, there were 30 studies involved in bacteriology of PTA during 1980–2016. The timeframes, the geographical locations and the predominant bacterial species identified in these studies were listed in Table 5.

**Table 5:** Studies involved in bacteriology of PTA during 1980–2016.

Investigator	Country	Year	Positive culture	Predominant aerobes	Predominant anaerobes
Brook et al. (1981)^19^	U.S.	–	16	*Gamma-hemolytic streptococci*	*Bacteroides* sp.
				*Alpha-hemolytic streptococci*	Anaerobic GPC
Jokipii et al. (1988)^8^	Finland	–	42	Group A *streptococcus*	*Peptostreptococcus* sp.
				*Streptococcus viridans* group	*Bacteroides* sp.
Brook et al. (1991)^37^	U.S.	1978–1985	34	*Staphylococcus aureus*	*Bacteroides* sp.
				*Streptococcus pyogenes*	*Peptostreptococcus* sp.
Snow et al. (1991)^38^	UK	–	55	*Beta hemolytic streptococci*	–
				*Staphylococcus aureus*	
Jousimies-Somer et al. (1993)^20^	Finland	–	122	*Streptococcus pyogenes*	*Fusobacterium necrophorum*
				*Streptococcus milleri* group	*Prevotella melaninogenica*
Mitchelmore et al. (1995)^9^	UK	1982–1992	45	Group A *streptococcus*	*Peptostreptococcus* sp.
					*Prevotella*
Muir et al. (1995)^39^	New Zealand	1990–1992	39	*Group A streptococcus*	–
Prior et al. (1995)^40^ Cherukuri (2002)	UK	–	45	–	–
	USA	1990–1999	82	*Streptococcus* sp.	–
				*Haemophilus* sp.	
Matsuda et al. (2002)^10^	Japan	1988–1999	386	*Alpha-hemolytic streptococci*	Anaerobic gram-negative rods
				*Neisseria* sp.	*Porphyromnas* sp.
Hanna et al. (2006)^41^	Northern Ireland	2001–2002	37	Group A *streptococci*	*Bacteroides* sp.
Sakae et al. (2006)^42^	Brazil	2001	26	*Streptococcus viridans*	*Peptostreptococcus* sp.
				*Streptococcus pyogenes*	*Prevotella* sp.
Zagolski et al. (2007)	Poland	–	12	*Streptococcus* sp.	*Bacteroides* sp.
Megalamani et al. (2008)	India	2003–2006	39	Beta hemolytic *streptococcus*	–
				Pseudomonas	
Sunnergren et al. (2008)^7^	Sweden	2000–2006	67	Group A *streptococcus*	*Bacteroides* sp.
Klug et al. (2009)^11^	Denmark	2001–2006	405	Group A *streptococcus*	*Fusobacterium* sp.
				Groups C or G *streptococci*	
Gavriel et al. (2009)^6^	Israel	1996–2002	137	*Streptococcus pyogenes*	*Prevotella* sp.
				*Streptococcus intermedius*	*Peptostreptococcus* sp.
Segal et al. (2009)^4^	Israel	2004–2007	64	Group A *streptococcus*	–
				Group C *streptococcus*	
Repanos et al. (2009)^32^	UK	1998–2005	107	*Streptococcal* sp.	–
Rusan et al. (2009)	Denmark	2001–2006	623	Group A *streptococcus*	*Fusobacterium* sp.
Acharya et al. (2010)^5^	Nepal	2007–2008	18	*Streptococcus pyogenes*	–
				*Staphylococcus aureus*	
Marom et al. (2010)^17^	Canada	1998–2007	180	*Streptococcus viridans*	–
				Group A *streptococcus*	
Hidaka et al. (2011)^43^	Japan	2002–2007	65	*Streptococcus milleri* group	*Prevotella* sp.
				Other *Streptococcus* sp.	*Peptostreptococcus* sp.
Klug et al. (2011)^12^	Denmark	2005–2009	36	*Streptococcus viridans*	*Prevotella* sp.
				*Neisseria* sp.	*Fusobacterium* sp.
Love et al. (2011)^13^	New Zealand	2006–2008	147	Group A *streptococcus* Other *beta-hemolytic streptococci*	*Fusobacterium* sp.
Albertz et al. (2012)^16^	Chile	2000–2012	112	*Streptococcus pyogenes*	*Bacteroides* sp.
				Other *streptococci*	*Peptostreptococcus* sp.
					*Fusobacterium* sp.
Takenaka et al. (2012)^3^	Japan	2005–2009	50	*Streptococcus pyogenes*	Anaerobic
					*Streptococcus*
					*Fusobacterium* sp.
Sowerby et al. (2013)^14^	Canada	2009–2010	42	Group A *streptococcus*	–
				*Streptococcus anginosus*	
Gavriel et al. (2015)	Israel	1996–2003	132	*Streptococcus pyogenes*	*Prevotella* sp.
					*Peptostreptococcus* sp.
Mazur et al. (2015)^18^	Poland	2003–2013	45	*Streptococcus viridans* group	*Fusobacterium* sp.
				*Streptococcus pyogenes*	*Prevotella* sp.
Plum et al. (2015)^44^	USA	2002–2012	69	*Streptococcus milleri* in adults	–
				β-hemolytic *streptococcus* in children	
Lepelletier et al. (2016)^36^	French	2009–2012	412	Group A *streptococci*	*Fusobacterium* spp.
Tachibana et al. (2016)^45^	Japan	2008–213	100	*Streptococcus viridans*	Fusobacterium sp.
Vaikjarv et al. (2016)^46^	Estonia	2011–2012	22	*Streptococcus* sp.	*Streptococcu*s spp.
Present study (2017)	Taiwan	1990–2013	168	*Streptococcus Viridans*	*Prevotella* sp.
				*Klebsiella Pneumoniae*	*Fusobacterium* sp.

–, indicates “not disclosed”.

Several broad-spectrum antibiotics such as penicillin or cefazolin combined with gentamycin (GM) and metronidazole, clindamycin plus GM, or augmentin along were used in our series. All these antibiotics were effective without any significant difference.

## Discussion

In our study, the most common pathogen identified through pus culturing in patients with PTA was *S. viridans*, followed by *K. pneumoniae*; commonly isolated anaerobes in our study were *Prevotella* and *Fusobacterium* spp. We reviewed the bacteriology data from previous studies, as shown in Table 5. Most of the studies^3^, ^4^, ^5^, ^6^, ^7^, ^8^, ^9^, ^10^, ^11^, ^12^, ^13^, ^14^, ^15^, ^16^ have reported group A *Streptococcus* as the most common aerobic pathogen in PTA; some studies^12^, ^17^, ^18^ have reported that common aerobic pathogens were *S. viridans*, followed by group A β-hemolytic *streptococci*. The prevalence of *K. pneumoniae* has been rarely reported in previous studies. In previous studies, *Fusobacterium nucleatum*,^3^, ^8^, ^11^, ^12^, ^15^, ^19^, ^20^ *Prevotella*,^3^, ^12^, ^19^, ^20^, ^21^ *Bacteroides*,^7^, ^8^, ^19^ *Peptostreptococcus*,^8^, ^9^, ^20^ and anaerobic *streptococcus*^12^ were the most common anaerobic pathogens. The divergence of bacterial culture may be owing to different geographical location. With difference between diets and lifestyle, the bacterial flora within each people may be also different.

*K. pneumoniae* and *Streptococcus* spp. are common oral flora normally found in the mouth and are odontogenic pathogens of deep neck infection.^22^, ^23^, ^24^ The *S. viridans* group is the etiological agent of dental caries, pericoronitis, or, if introduced into the bloodstream, endocarditis. In Taiwan, *K. pneumoniae* has been linked to lung infection in aspiration patients or a liver abscess^25^ in immunocompromised patients or those with diabetes.^26^

Patients with old age^27^ or diabetes mellitus^28^ are considered to be immunocompromised and have more chance to get infection. DM and elder are also linked with more complications and higher mortality rate in deep neck infection.^29^, ^30^ Thus PTA patients with above characteristics often have longer hospital stay.^30^ We reported the microbiology of PTA in such immunocompromised patients. Patients with DM had no increased risk of isolating *K. pneumoniae* as the causative pathogen of PTA. By contrast, elderly patients with PTA in the current series had a higher risk of *K. pneumoniae* isolation.

A trend toward a higher isolation rate for anaerobes was observed during 2002–2013 (*p* = 0.048). Gavriel^6^ reported a significant increase in anaerobic growth during 1996–1999 and then a slow nonsignificant decline until 2002. Takenaka^3^ reported no change in the percentage of cases with anaerobic growth between 2 periods (2005–2007 and 2008–2009). Such a phenomenon might result from a real change in pathogens; the alteration of antibiotics used, or improved culture methods for anaerobic pathogens. In our series, no major alteration of antibiotics used or improvement of the culture methods was observed. Physicians should prescribe empirical antibiotics to cover anaerobes.

PTA is often a polymicrobial infection. Polymicrobial growth was observed in the pus cultures of 57.39% of patients. The rationale of using empirical antibiotics was to cover GPCs, GNBs, and respiratory anaerobes. If necessary, suitable antibiotics should be chosen on the basis of culture results. However, the management of most uncomplicated patients may not be affected by the culture result.^31^ Repanos et al.^32^ suggested that using broad-spectrum antibiotics such as cephalosporin or penicillin combined with metronidazole was effective. In our study, no significant difference was found among several combinations of broad-spectrum antibiotics.

Smoking habit has been commonly observed in patients with PTA in several studies^17^, ^18^, ^33^, ^34^; these studies have reported smoking as a risk factor for PTA. Marom et al.^17^ reported a significantly higher incidence for *S. viridans*, other gram-positive cocci isolates, and anaerobes. In our study, no statistical significance was observed in the causative pathogen between smokers and nonsmokers with PTA, similar to the findings of the study by Klug.^34^

Betel nut chewing is a popular habit in Southeast Asia. To the best of our knowledge, no study has found an association between the bacteriology of PTA and betel nut chewing. In our series, this habit was associated with a higher risk of GPC as a pathogen. In the study by Ling et al.,^35^ it was associated with a likelihood of subgingival infection by *Actinobacillus actinomycetemcomitans* and *Porphyromonas gingivalis.*

In our study, elderly patients (older than 65 year-old) had a high risk of *K. pneumoniae* isolation. The study by Marom^17^ reported a significantly higher isolation rate for infection by GPC (mixed *Streptococcus* species) and gram-negative rods in older patients (40 year-old or older) than in younger patients.

The hot season increased the risk of GPB infection and reduced the risk of *K. pneumoniae* infection in patients with PTA in our current study. Our institute is located in a tropical region that has approximately six months (May to October) of hot weather, with a mean temperature of 27 °C. By contrast, Klug et al.^15^ from another institute located in a temperate zone reported a higher incidence of *F. nucleatum* infection during summer than during winter. It also reported Group A *streptococcus* was significantly more frequently identified from in the winter and spring. The study in French^36^ reported PTA caused by *S. pyogenes* or anaerobes were more prevalent in the winter and spring than summer. Such fluctuation in the microbiology of PTA might be weather related.

In our series, no specific microorganism was associated with the poor prognosis of PTA. This finding is considerably similar to the reports by Marom^17^ and Mazur.^18^

Our study has several limitations. Because we retrospectively collected data by chart review, data from the medical record might be lost during the early years. As we used several small populations of isolated pathogens, a larger sample size is necessary to determine the relationship between the isolated pathogen and the predisposing factors.

## Conclusions

The most common causative pathogen of PTA was *S. viridans*, followed by *K. pneumoniae*. The isolation of anaerobes significantly increased in recent years. The common ones were *Prevotella* and *Fusobacterium* spp. Empirical antibiotics targeting both aerobes and anaerobes should be appropriate as treatment. Bacterial isolation may be associated with host clinical factors, environmental factors, and hospitalization duration.

## Conflicts of interest

The authors declare no conflicts of interest.
